# Determinants of Transmission Risk During the Late Stage of the West African Ebola Epidemic

**DOI:** 10.1093/aje/kwz090

**Published:** 2019-04-03

**Authors:** Alexis Robert, W John Edmunds, Conall H Watson, Ana Maria Henao-Restrepo, Pierre-Stéphane Gsell, Elizabeth Williamson, Ira M Longini, Keïta Sakoba, Adam J Kucharski, Alhassane Touré, Sévérine Danmadji Nadlaou, Boubacar Diallo, Mamamdou Saidou Barry, Thierno Oumar Fofana, Louceny Camara, Ibrahima Lansana Kaba, Lansana Sylla, Mohamed Lamine Diaby, Ousmane Soumah, Abdourahime Diallo, Amadou Niare, Abdourahmane Diallo, Rosalind M Eggo

**Affiliations:** 1 Department of Infectious Disease Epidemiology, London School of Hygiene and Tropical Medicine, London, United Kingdom; 2 World Health Organization, Geneva, Switzerland; 3 Department of Medical Statistics, London School of Hygiene and Tropical Medicine, London, United Kingdom; 4 Department of Biostatistics, University of Florida, Gainesville, Florida; 5 World Health Organization Ebola Vaccination Team, Conakry, Guinea; 6 Ministry of Health, Conakry, Guinea

**Keywords:** Ebola, Guinea, multiple imputation, regression analysis, risk factors

## Abstract

Understanding risk factors for Ebola transmission is key for effective prediction and design of interventions. We used data on 860 cases in 129 chains of transmission from the latter half of the 2013–2016 Ebola epidemic in Guinea. Using negative binomial regression, we determined characteristics associated with the number of secondary cases resulting from each infected individual. We found that attending an Ebola treatment unit was associated with a 38% decrease in secondary cases (incidence rate ratio (IRR) = 0.62, 95% confidence interval (CI): 0.38, 0.99) among individuals that did not survive. Unsafe burial was associated with a higher number of secondary cases (IRR = 1.82, 95% CI: 1.10, 3.02). The average number of secondary cases was higher for the first generation of a transmission chain (mean = 1.77) compared with subsequent generations (mean = 0.70). Children were least likely to transmit (IRR = 0.35, 95% CI: 0.21, 0.57) compared with adults, whereas older adults were associated with higher numbers of secondary cases. Men were less likely to transmit than women (IRR = 0.71, 95% CI: 0.55, 0.93). This detailed surveillance data set provided an invaluable insight into transmission routes and risks. Our analysis highlights the key role that age, receiving treatment, and safe burial played in the spread of EVD.

Between December 2013 and June 2016, the largest Ebola virus disease (EVD) epidemic to date occurred in West Africa, causing more than 28,000 cases, mainly in Liberia, Sierra Leone, and Guinea (1). Of these, 3,804 confirmed cases and 2,536 deaths were in Guinea (1). There remains a pressing need to understand the transmission dynamics of this outbreak, so that interventions can be designed and accurate forecasts made as outbreaks continue to occur (2, 3).

During the 2013–2016 epidemic in Guinea, intensive epidemiologic investigation was made of cases, including assembling individuals into chains of transmission, which link infected individuals to their descendant cases during case investigations (4). In contrast to studies of cases, which can give insight only into risk factors for acquisition of EVD (4–6), or genetic analysis, which has been used to reconstruct spatial dispersion of the disease in different regions (7), transmission chains allow detailed analyses of the risk factors for onward transmission (8, 9). These data are invaluable for understanding the characteristics of individuals likely to have high onward transmission, but to our knowledge, they have been underused in analyses of this epidemic.

Using a large database of epidemiologically reconstructed transmission chains, we summarized information on cases reported in the late stages of the 2013–2016 epidemic in Guinea. We used this information to identify characteristics of cases that were associated with increased onward transmission.

## METHODS

Data from 2 databases were linked in this study: the transmission-chain data set (1,012 cases) and the Guinean surveillance database of EVD cases. A group of 152 individuals in the transmission-chain data set were participants in the *Ebola ça Suffit* ring vaccination trial (10, 11). These cases were excluded from this study because of the likely impact of the trial on transmission, leaving 860 cases used in this analysis.

### Transmission-chain data set

Data on cases and the epidemiologic links between them were collected by the Ministère de la Santé et de l’Hygiène Publique of Guinea (Ministry of Health and Public Hygiene) during the epidemic. Field teams conducted interviews with cases (where possible) and their contacts, as part of epidemiologic investigations. Based on contact with confirmed or probable cases, the most likely infector or infectors were assigned to each case. The chains of transmission were continually revised and updated during the EVD response in Guinea, and when new cases were confirmed, those were added to the database and to the chains. This could result in changes to the likely infector or joining subtrees together as new information became available. The chains therefore represent the best possible epidemiologic linkage of cases to each other, made by trained field teams with access to cases, contacts, and contextual information. We restricted our analysis to confirmed and probable cases infected between September 2014 and November 2015, because transmission chains were available during this time period, and resources were available to digitize these data.

Variables were age, sex, location (prefecture, subprefecture, and village), survival status of the case, whether the burial was safe or unsafe, the epidemiologically inferred source of infection, and the route of transmission (including household, nosocomial, neighbor, Ebola treatment unit (ETU)), as well as national identification number for each individual (Table 1). Geographic information, demographic variables, and the probable routes of transmission were ascertained by the field epidemiology teams (Web Appendix 1, available at https://academic.oup.com/aje). Safe burial means that burial was safe, dignified, and conducted by a trained burial team. We used dates of: 1) onset, 2) admission to an ETU, 3) discharge from an ETU, 4) death, and 5) burial. We deleted 10 implausible epidemiologic links (for example, where the end of symptoms of the infection recipient was earlier than the start of symptoms of the named infection transmitter). When several infection transmitters were reported and plausible for a case, we considered only 1 link in the transmission chain by random selection. We conducted sensitivity analysis on this selection.

**Table 1.** Characteristics of the Data Set Before Imputation (*n* = 818), Ebola Epidemic, Guinea, 2014–2016

Variable	No. of Cases	%	Transmission Status (%)		
			None	Moderate	High
Age group, years					
0–14 (children)	137	15.9	88.3	10.2	1.4
15–99 (adults)	621	72.3	64.1	27.3	8.7
Unknown	102	11.8	41.2	51.9	6.9
Sex					
Male	391	45.5	69.6	24.3	6.2
Female	454	52.8	62.3	29.3	8.3
Unknown	15	1.7	40.0	60.0	0
EVD status					
Confirmed	661	76.9	72.3	23.4	4.2
Probable	199	23.1	41.7	41.2	17.1
Number of reported infectors					
First generation of a chain	133	15.4	31.6	55.6	12.8
1 infector	690	80.2	71.0	22.8	6.2
2 infectors	16	1.9	68.8	25.0	6.2
3 infectors	21	2.5	85.7	9.5	4.8
Route of infection^a^					
Household transmission	217	75.9			
Nosocomial transmission	30	10.5			
Funeral transmission	36	12.6			
Other transmission	74	26.2			
Outcome					
Survivor, ETU+	235	27.3	85.5	13.2	1.3
Nonsurvivor, ETU+, safe burial	232	27.0	71.6	24.6	3.9
Nonsurvivor, ETU−, safe burial	63	7.3	57.1	38.1	4.8
Nonsurvivor, ETU−, unsafe burial	70	8.1	35.7	40.0	24.3
Unknown	260	30.2	51.2	37.3	11.5
Location					
Rural area	458	53.3	60.0	31.9	8.1
Urban area	402	46.7	71.1	22.6	6.2

Abbreviations: ETU+, attended an Ebola treatment unit; ETU−, did not attend an Ebola treatment unit.

^a^ More than 1 route was specified for some cases, so we did not compute the transmission status for this variable; 286 cases caused transmission.

### Guinea surveillance database

The Guinean surveillance database is a line list of confirmed cases in Guinea from the national surveillance system. Each record contains the same information on each case as the transmission-chain data set, except for the transmission link, but completeness of other fields (such as dates) is higher. Therefore, we matched the transmission-chain data set to the surveillance database using national identification number, or name, location, age, and dates of infection. This increased the completeness of the data used in this analysis.

### Matching cases

A total 664 cases in the transmission-chain data set (77.2%) matched with a record in the surveillance database; 135 (15.7%) of these did not provide additional information on the case. We compared the features of the 529 remaining cases in each database to eliminate mismatches (Web Appendix 2, Web Figures 1 and 2) and used the surveillance database to supplement features of 380 (44.2%) cases. Among these cases, all reported variables matched for 71 individuals, and the surveillance database contributed information for 380 cases. In cases that were not perfectly matched, the mismatches were minor, and we assumed these differences were due to data-entry errors given that other variables matched (Web Appendix 2). For the other cases, we kept the features described in the transmission-chain data set. Of the 860 individuals in the transmission-chain data set, 196 could not be matched to cases in the Guinean surveillance database (22.8%). Table 1 shows reporting and values of each variable in the final data set.

### Classification by number of transmissions

We calculated the number of reported secondary cases for each individual, and we categorized them as: 1) high transmitters (more than 3 cases), 2) moderate transmitters (1–3 cases), or 3) no onward transmission. We tested for associations with demographic characteristics (Web Appendix 3, Web Figure 3).

### Statistical analysis

We used negative binomial regression to estimate the impact of characteristics of the cases on the number of secondary cases caused (12). We grouped the age of cases into categories, in years, of 0–14, 15–34, 35–54, 55–74, and ≥75. Conakry and prefecture city centers were considered to be urban areas, and other areas (villages, towns) were defined as rural areas. Cases without a known infection transmitter were defined as the first generation of a chain and all others as subsequent generations of a chain.

We created a variable that combined the survival status, ETU attendance, and burial status of each case, called the “outcome.” In the data, all reported survivors had been admitted to an ETU, and all nonsurvivors that had been admitted to an ETU had a safe burial. Therefore we used 4 unordered levels describing the outcome of each case: 1) survivor who attended an ETU, 2) nonsurvivor who attended an ETU and was safely buried, 3) nonsurvivor who did not attend an ETU and was safely buried, 4) nonsurvivor who did not attend an ETU and was unsafely buried (Web Appendix 3, Web Figure 4).

### Imputation of missing data

Four of the variables included in the negative binomial regression analysis were incompletely reported: sex (1.7% missing), age (11.8% missing), survival status (4.5% missing), burial safety status (29.4% missing), and ETU admission status (13.6% missing) (Web Appendix 3, Web Figures 5 and 6). Because some of the variables were incomplete, and we aimed to retain the full population in the regression analysis, we used multiple imputation for missing values (13–15). In the imputation model, we considered all factors included in the regression analysis as explanatory variables (see below) as well as 4 others from the database: 3 for the number of transmissions caused (community, funeral, or nosocomial) and one for month of onset. The imputed variables were age (using predictive mean matching), survival status, burial safety status, and ETU admission status (logistic regression). We assumed that missing data were missing at random (16) (Web Appendix 4, Web Table 1, Web Figures 7 and 8). Forty data sets were generated using the MICE package in R, version 3.5.0 (R Foundation for Statistical Computing, Vienna, Austria) (17). We used pooled coefficient estimates drawn from 40 imputed data sets (Web Appendix 4, Web Table 2, Web Figure 9), and we performed sensitivity analysis on the multiple imputation (Web Appendix 5, Web Tables 3 and 4).

## RESULTS

### Chains of transmission

**Figure 1.**
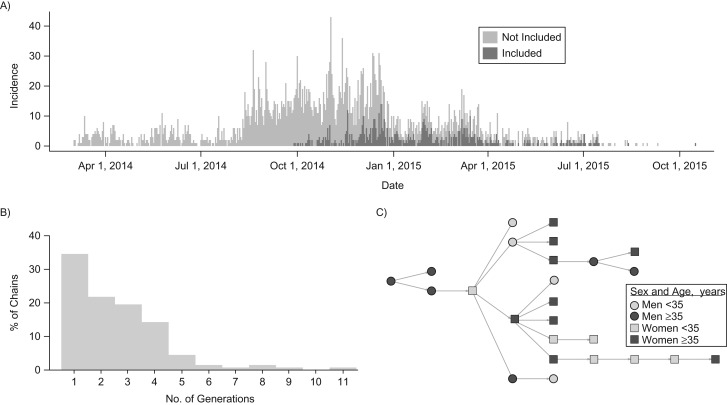
Characteristics of the chains data, Ebola epidemic, Guinea, 2014–2016. A) Time series of the daily incidence in Guinea. Light gray is total incidence, and dark gray area shows cases included in this analysis. B) Distribution of the number of generations per chain. C) Example of a chain with 9 generations. Squares symbolize women, and circles symbolize men. Lighter shade is under 35 years of age, and darker shade is over 35 years of age.

The proportion of the total cases represented in this data set increases through time (Figure 1). There were 818 cases in 87 chains of transmission of 2–11 generations (Figure 1) and 42 individuals not linked to any infector or to subsequent cases. These first-generation cases occurred throughout the study period (Web Figure 2). The largest chain of transmission included 78 cases, starting on January 1, 2015, and ending on April 25, 2015.

**Figure 2.**
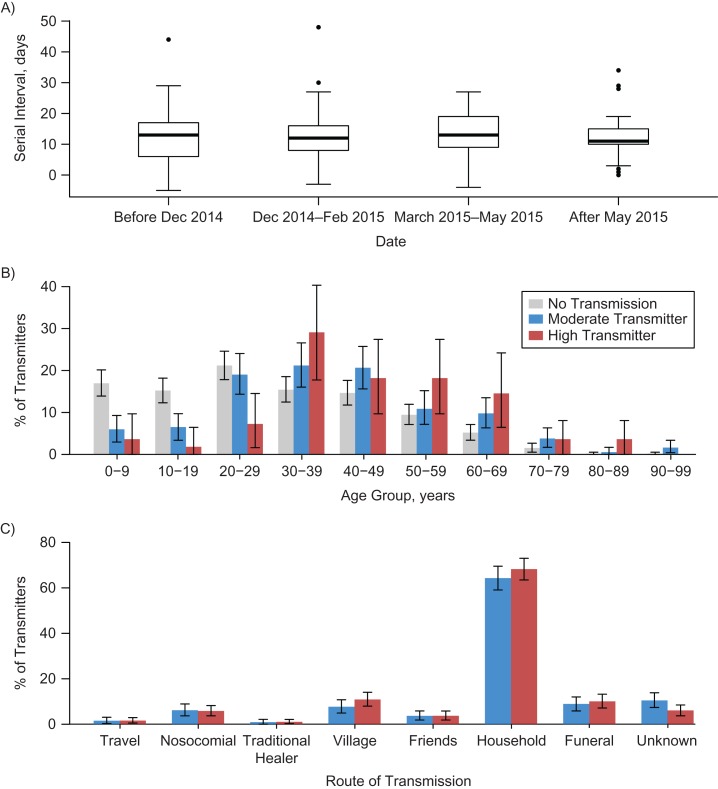
Characteristics of transmission, Ebola epidemic, Guinea, 2014–2016. A) Serial interval through time (overall mean, 12.3 days). Number of cases in each group: 61, 163, 61, and 23, respectively. B) Distribution of the transmitter status of the cases depending on the age of the cases. C) Distribution of the transmitter status depending on the route of transmission.

The mean serial interval (time between the date of onset of the infected case and onset in the person who transmitted the infection) was 12.3 days (Figure 2A), calculated from 308 serial intervals. The serial interval did not vary through time (Figure 2A), by route of transmission, by age, or by generation of the chain (Web Appendix 6, Web Figure 10).

### Reproduction number

**Figure 3.**
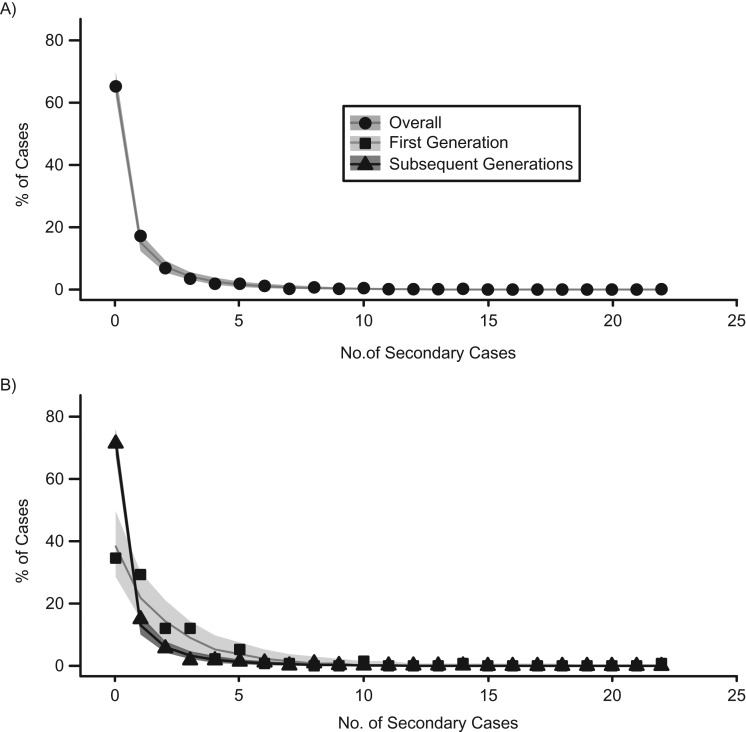
Distribution of secondary cases, Ebola epidemic, Guinea, 2014–2016. A) Distribution of the number of secondary cases per case, fitted to a negative binomial distribution (line and shaded areas). B) Fits of the first or the subsequent generations only (total: mean, 0.89 (standard deviation, 0.31; 95% confidence interval: 0.25, 0.37); first generation: mean, 1.77 (standard deviation, 0.88; 95% confidence interval: 0.53, 1.23); subsequent generations: mean, 0.70 (standard deviation, 0.25; 95% confidence interval: 0.19, 0.30)).

The unadjusted average number of secondary cases per individual was 0.89. Most cases did not result in subsequent transmission. A total of 299 (34.8%) cases resulted in at least some transmission, and 62 (7.2%) individuals were deemed high transmitters and were responsible for 53.5% of the transmission events observed. The maximum number of observed secondary cases was 22. We fitted a negative binomial distribution to the number of secondary cases and found high dispersion (mean, 0.89; dispersion parameter, 0.31 (95% confidence interval (CI): 0.25, 0.37); index of dispersion, 3.87 (95% CI: 3.41, 4.56)). We stratified first and subsequent generations and observed a higher reproduction number among first-generation individuals (mean = 1.77) than among the subsequent generations (mean = 0.70) (Figure 3, Web Appendix 6, Web Figure 11).

### Univariable description of transmitters

Older cases were more likely to be high transmitters, whereas children or young adults were less likely to transmit; 49.4% of the cases who did not cause any transmission were younger than 30, whereas only 11.3% of high transmitters fell into this age category (Figure 2B). We did not observe any change in the route of transmission between moderate and high transmitters (Figure 2C). The major route of transmission was through the household (57.9% of all cases and 75.9% of transmission events). We did not observe any difference between case characteristics (such as sex and location) and classification of high, moderate, or no transmission (Web Appendix 3, Web Figure 3).

### Determinants of transmission

We found significant associations with the number of secondary cases generated of the following characteristics: sex, outcome (4 unordered levels), age category, and being the first generation of a chain (Table 2). In our multivariable negative binomial regression model, the estimated intercept was 0.69 (95% CI: 0.38, 1.28) and was defined as the mean number of secondary cases for women, aged 35–54, who did not survive, did not go to an ETU, and had a safe burial, in an urban area, and who were not the first generation of a chain (Table 2).

**Table 2.** Results of Regression Analysis to Evaluate Associations With Number of Secondary Cases Generated, Ebola Epidemic, Guinea, 2014–2016

Variable	Secondary Cases	IRR	95% CI	*P* Value
Intercept^a^	0.69		0.38, 1.28	0.239
Sex				
Female		1.00	Referent	
Male		0.71	0.55, 0.93	0.012
Outcome				
Alive, ETU+		0.31	0.19, 0.51	<10^−3^
Dead, ETU+, safe burial		0.62	0.38, 0.99	0.046
Dead, ETU−, safe burial		1.00	Referent	
Dead, ETU−, unsafe burial		1.82	1.10, 3.02	0.018
Location				
Urban		1.00	Referent	
Rural		1.18	0.90, 1.54	0.224
Age group, years				
0–14		0.35	0.21, 0.57	<10^−3^
15–34		0.68	0.49, 0.93	0.015
35–54		1.00	Referent	
55–74		0.94	0.63, 1.40	0.757
75–99		1.47	0.55, 3.91	0.438
Generation				
First		1.76	1.27, 2.44	0.001
Subsequent		1.00	Referent	

Abbreviations: CI, confidence interval; ETU+, attended an Ebola treatment unit; ETU−, did not attend an Ebola treatment unit; IRR, incidence rate ratio.

^a^ Defined as the mean number of secondary cases for women, aged 35–54 years, who did not survive, did not go to an ETU, and had a safe burial, in an urban area, and who were not the first generation of a chain.

Individuals younger than 35 years caused significantly fewer secondary cases, and the first generation of chains caused significantly more secondary cases than subsequent generations (incidence rate ratio (IRR) = 1.76 (95% CI: 1.27, 2.44)). Men caused significantly fewer secondary cases than women (IRR = 0.71 (95% CI: 0.55, 0.93)), which was not observed in the univariable analysis.

By comparing the coefficients for nonsurvivors who did and did not attend an ETU, but had safe burials, we determined that there was a significantly lower number of secondary cases among those who attended an ETU (IRR = 0.62 (95% CI: 0.38, 0.99)). We found that unsafe burial was associated with a large increase in transmission (IRR = 1.82 (95% CI: 1.10, 3.02)). We compared individuals who survived with nonsurvivors among those who attended an ETU and had a safe burial, and we found that survival was associated with significantly fewer secondary cases than nonsurvival (IRR = 0.51 (95% CI: 0.31, 0.82)) (Web Table 3).

There was no significant association between urban or rural location and number of subsequent cases.

## DISCUSSION

Using the largest set of epidemiologically linked transmission trees available for EVD, we identified key patient characteristics associated with increased onward transmission and estimated their association with the number of secondary cases each case generated. By doing this, we have been able to quantify the association between attending an ETU and safe burials on onward transmission in the late stage of the epidemic in Guinea.

Attending an ETU was associated with a large decrease in the number of transmission events, and unsafe burial was associated with an almost 2-fold increase in number of transmissions. Our estimates emphasize the importance of ETU attendance and safe burials as control measures for Ebola, and are similar to values found in other studies (8, 18–22). These data are drawn from the late stage of the epidemic, and the same risk factors for transmission extend even late in the epidemic, when awareness of EVD transmission routes might have been higher.

In agreement with previous studies of EVD transmission, we found a highly skewed distribution of secondary cases (18, 19, 23, 24). Indeed, the majority of cases did not transmit EVD at all, and only a small number had high numbers of transmission events (25). Importantly, we were able to determine the case characteristics associated with the number of transmissions. This information could be used in real-time prediction, by incorporating information on the case mix of incident cases.

Our analysis is of disjoint transmission chains, which are observations of a fully connected transmission tree. This complete tree contains the entire outbreak, with each case linked together. Our findings relating to the first generation of each chain are therefore a measure of the impact of a case not having a traced link to a prior case and not the absence of a true link to a prior case.

We found that the first generation of each chain was associated with a higher number of secondary cases than those identified later in the chain. The first generation of each chain is necessarily an individual who could not be epidemiologically linked to any prior chains by the field epidemiology teams. First-generation cases might have spent longer in the community (and therefore had a longer transmission window) because either they were not traced by contact-tracing and therefore did not know they were at risk (26) or they evaded contact-tracing (27). Alternatively, or additionally, there might be a bias toward detection of large transmission events in our data, whereby untraced contacts are more likely to be detected if they give rise to a larger cluster of cases (i.e., ascertainment bias).

We found that children and young adults had lower onward transmission, whereas infections in older adults were more likely to result in large numbers of secondary cases. Several studies have determined that children were at lower risk of infection in previous outbreaks (6, 28) and during the West African epidemic (4, 5, 29); behavioral differences in caring roles are suggested as the reason (28–30). Our study adds to evidence (31, 32) that children were also associated with lower risk of onward transmission, although not all analyses find this pattern (33).

Three quarters of reported transmissions were in the household, making it the most frequent transmission route. Studies from the early stages of the epidemic found a higher contribution from funeral and nosocomial transmission routes (8) than we observed here. Our findings could be the result of public health interventions to increase safe and dignified burials, protect health-care workers, and raise awareness of transmission risks (22, 34).

There were several interventions occurring at the time of the study (11, 18). We accounted for the largest of these—the *Ebola ça Suffit* ring vaccination trial—by removing participants in the trial. Other interventions could have affected transmission, although we did not detect an association between time and the number of secondary cases in the model. In addition, the study period is in the latter part of the epidemic, and there might be differences in inferred transmission risk in other time periods of the epidemic.

Although we did not find evidence for differences between cases in the transmission-chain data set and those who were not, it is possible that there are different characteristics in the number of secondary cases generated.

This study is limited to observed cases, and therefore the number of transmission events could be lower than the true value. Of note, there were no survivors who did not attend an ETU in our data. It is likely that these survivors remained undetected, and therefore we could not include transmission risk from these individuals in our analysis. Not attending an ETU could be associated with other transmission risks or with community resistance to interventions (35).

Multiple imputation provides unbiased estimates under the assumption that missing data were missing at random: Given all the information available, the missing values were similar in distribution to the observed values. If, for example, all individuals with an unknown burial status in fact had an unsafe burial, then this assumption might be violated, potentially leading to bias in the estimated regression coefficients.

Some of the links inferred by on-the-ground epidemiologists might be incorrect, which could affect our estimates of determinants of onward transmission. However, in contrast to other studies, which retrospectively linked cases into transmission chains (31, 36), the chains used in our study were generated in real time. Genetic data linking cases together could be used to test whether there are incorrect links (37), but these data were not available for this study.

By the end of the epidemic, the chains of transmission represented the best possible record of epidemiologic investigations of EVD cases in Guinea. During periods of high numbers of cases, the epidemiologic teams might have been more stretched and therefore surveillance effort per case could have been lower. It is possible that proposed infection transmitters have been misspecified during this time period, which could affect the findings. However, based on the characteristics of cases, we think it is unlikely that this would be a systematic error.

During the EVD outbreak in Guinea, detailed investigations were conducted around each case reported to surveillance in order to inform the public health response. This enormous undertaking resulted in large quantities of data that provided invaluable insight into the routes and risk of transmission. Recent outbreaks in the Democratic Republic of the Congo indicate the vital importance of epidemiologically informed measures in the control of Ebola (38, 39). Analyses of these data reveal the key role that older individuals and those that did not seek treatment played in the spread of EVD.

## Supplementary Material

Web Material

## Abbreviations

CI: confidence interval
ETU: Ebola treatment unit
EVD: Ebola virus disease
IRR: incidence rate ratio
