# Supplementary material for: Determinants of Transmission Risk During the Late Stage of the West African Ebola Epidemic
Source: Am J Epidemiol. 2019 Apr 3;188(7):1319–27. doi: 10.1093/aje/kwz090 (PMC6601535; doi:10.1093/aje/kwz090)
Supplement: Web Material [file kwz090_eggo_web_material_final.pdf]

Web Material

Determinants of transmission risk during the late stage of the West African Ebola epidemic

Alexis Robert, W. John Edmunds, Conall H. Watson, Ana Maria Henao-Restrepo, Pierre-Stéphane Gsell, Elizabeth Williamson, Ira M. Longini Jr., Keïta Sakoba, Adam J. Kucharski, Alhassane Touré, Sévérine Danmadji Nadlaou, Boubacar Diallo, Mamamdou Saidou Barry, Thierno Oumar Fofana, Louceny Camara, Ibrahima Lansana Kaba, Lansana Sylla, Mohamed Lamine Diaby, Ousmane Soumah, Abdourahime Diallo, Amadou Niare, Abdourahmane Diallo, and Rosalind M Eggo

Web Appendices 1-6; Web Figures 1-11; Web Tables 1-4

Web Appendix 1 - Description of the transmission chain dataset

a. Cases included in the analysis

The transmission chain dataset describes epidemiologically-linked confirmed and probable cases, infected in the latter stages of the 2013-2016 outbreak. Symptoms of the earliest case in the database were reported on 28 September 2014. Out of the 860 cases included in the dataset, 46 are not connected to an index nor to subsequent cases. 87 chains of transmission including between 2 and 78 individuals are listed. We removed 10 incoherent links where the end of symptoms of the infectee was reported after the start of symptoms of the infector.

b. Further information on variables

Age, gender, location (prefecture, sub-prefecture and village) were collected at the time of notification of the case. We used the prefecture and sub prefecture to classify case location into urban and rural: every individual from Conakry prefecture or from a sub prefecture labelled “Centre” was classified as urban, those remaining were classified as rural.

The dates of onset, admission to an ETU, discharge, death and burial are recorded in both the surveillance and chains database, although they were not complete in either. The survival status of each case is also recorded in both datasets.

A burial was classified as safe if it was a safe and dignified burial conducted by a trained burial team, and unsafe otherwise. Increasing the proportion of burials by trained teams remains a cornerstone of EVD control efforts, and is reported in WHO situation reports for outbreaks and epidemics (1).

The epidemiologically-inferred source of infection was determined by field epidemiologists. The field teams conducted interviews with cases (where possible) and their contacts, as part of epidemiological investigations. Based on contact with confirmed or probably cases, the most likely infector or infectors were assigned to each case. The chains of transmission were continually revised and updated during the EVD response in Guinea, and when new cases were confirmed those were added to the database and to the chains. This could result in changes to the likely infector or joining sub-trees together as new information became available. The chains therefore represent the best possible epidemiological linkage of cases to each other, made by trained field teams with access to cases, contacts, and contextual information.

The route of transmission between index and case is classified as nosocomial, funeral, friends, traditional healer, travel, household, village and unknown transmission, these categories are not exclusive. The route of transmission was assigned by the same field teams according to how contact was made with the most likely infector. In the case of multiple possible infectors, information was recorded on each, and we provide a sensitivity analysis on which infector is used in Web Appendix 5.

The national identification number is assigned at the time of entry into the surveillance database.

c. Removal of cases in the Ring Vaccination trial

We matched individuals in the RVT database to the transmission chain dataset, in order to determine their RVT participation status. RVT participants were matched by first name, last name, age, location, and date of infection by hand, and then removed from the analysis. Out of the 1012

cases first listed in the transmission chain dataset, 152 were part of the RVT. We removed these individuals and included the 860 remaining cases in the analysis.

Web Appendix 2 - Matching datasets

a. Surveillance database

The Ministry of Health of Guinea maintained a database of all confirmed and probable EVD cases. Transmission links were not reported in the Guinean surveillance database, however description of the case was more complete. Therefore, we matched individuals in the transmission chain dataset to the Guinean surveillance database to maximise information on each case and their links.

We found a potential match for 664 of the 860 cases. For 135 of them, the surveillance database did not provide any extra information. For the remaining 529 cases, we needed to assess the reliability of the match and exclude any potential mismatches. If a match was assessed as reliable, we described the individual using the features reported in the surveillance database, otherwise we kept the description from the transmission chain dataset. We built the algorithm presented in Web Figure 1. When national ID was reported in the transmission chain dataset, we only verified the prefecture to validate the match. Otherwise, we looked for differences in the case description. We aimed to minimise the chances of mismatch, whilst integrating the possible typos or mistakes from data collection, hence we accepted age difference lower than five years, and we used the Jaro-Winkler distance to compare first and last names. We also compared the reported management of the case (potential admission to an ETU). For the 81 remaining cases, we checked the timing of the disease in the surveillance database, in regard of the timing of the reported index and subsequent cases.

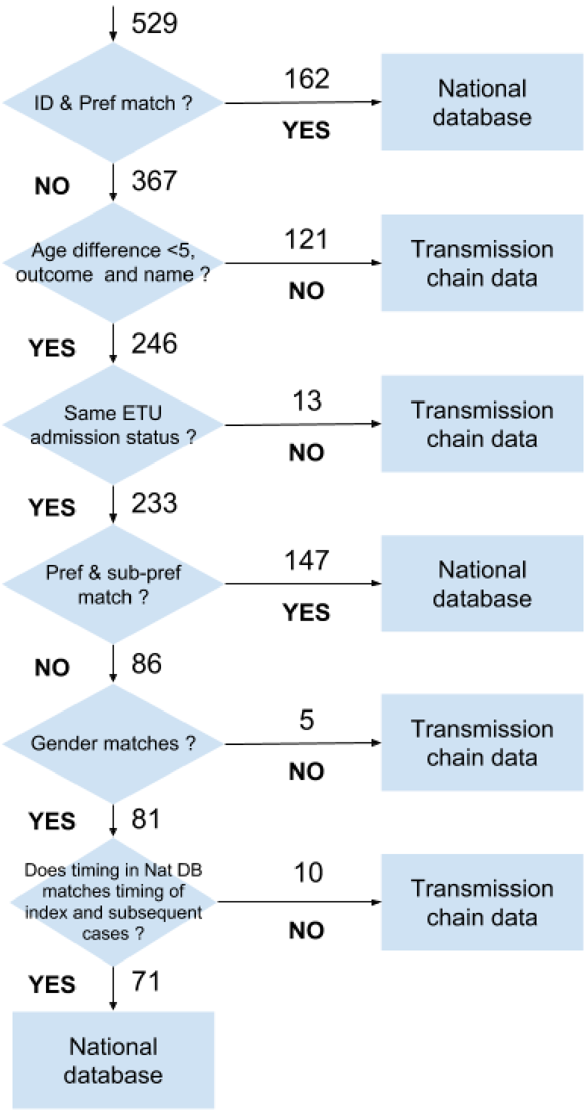

Web Figure 1: Decision tree used for comparing features from the surveillance database and the transmission chain dataset, and creating the final dataset

Overall, we used the surveillance database to supplement information for 380 cases. For 71 of them, all the reported information (timing of the disease, age, location, gender, ETU and burial status, national ID, name or outcome) matched. Hence the surveillance database was used to add new information unreported in the transmission chains. Regarding the other matches, we observed minor differences in location (42 cases had different sub-prefectures), age (61), name (111), timing (84) or survival status (14). Name mismatches were small differences in spelling or typing, or use of a middle or nick name, and not differences in whole name. For those cases with one or several mismatching variables, the values in both datasets were close and other variables matched. Therefore, we considered the differences were due to mistakes during data collection or data entry. We followed the decision tree in Web Figure 1 to decide whether a match was considered reliable.

b. Comparison of surveillance database and transmission chain cases in 2014

As displayed in the Main text (Figure 1A), the fraction of cases from the surveillance database included in the transmission chain dataset in 2014 was low. This fraction increased in 2015. We compared some features of the 2014 cases from the transmission chain dataset to the cases included in the surveillance database in 2014, and checked for selection bias. The distributions were compared using Kolmogorov-Smirnov test.

51% of the cases in the transmission chain dataset were women, there were 49% of women in the surveillance database (pvalue = 1). There were no differences in the age distribution: the mean age in each dataset was 39 years (pvalue = 0.96). 33% of cases in 2014 did not survive in the transmission chain dataset, 34% in the surveillance database in 2014 (pvalue = 1). Comparison of the distributions did not highlight any difference or selection bias for the cases included in the transmission chain dataset in 2014.

Web Figure 2 shows the onset date of the indexes of a chain through time, showing that chains started throughout the epidemic. Indexes of a chain represent individuals for which the field teams were not able to identify their potential infector.

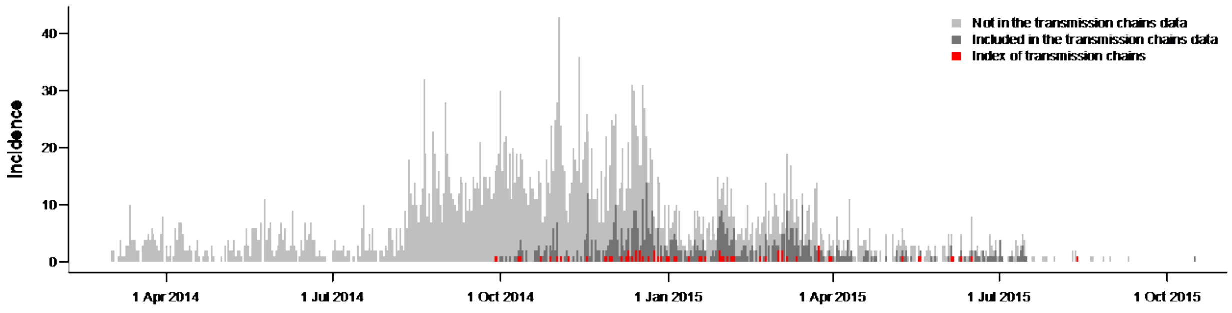

Web Figure 2: Time series of the daily incidence in Guinea (as figure 1a in the main text) showing the onset dates of the transmission chain indexes in red.

Web Appendix 3 - Variable creation and processing

a. Number of transmission events

We explored the distribution of individuals according to their transmission classification (high, moderate, no transmission) in the dataset. We did not observe any influence of gender (Student t test: p = 0.78, Web Figure 3A), or location (Student t test: p = 0.84) on the number of secondary cases (Web Figure 3B).

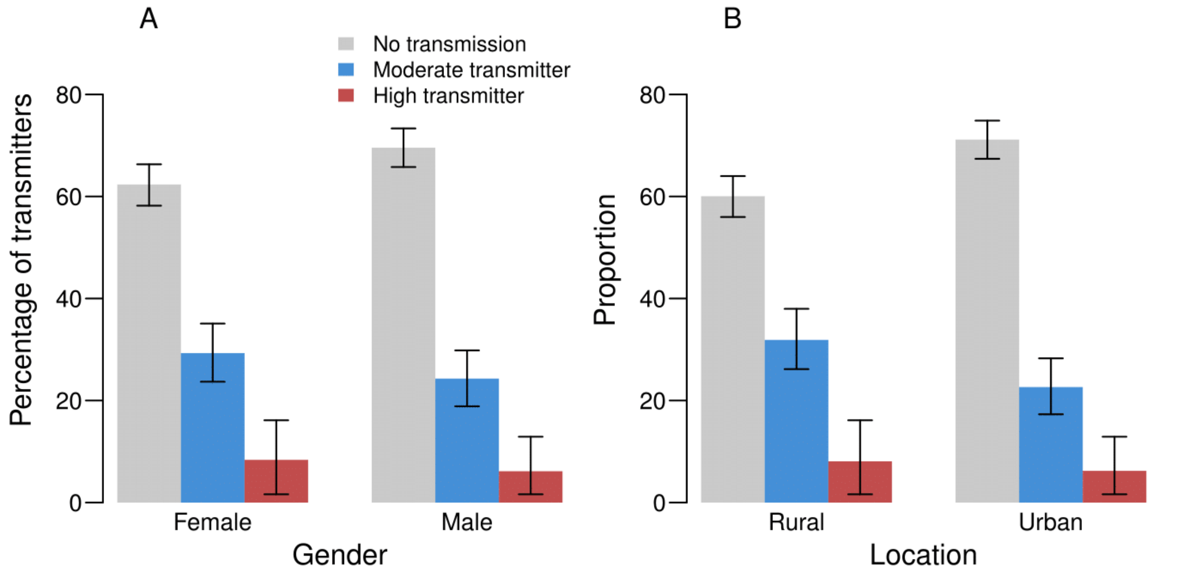

Web Figure 3. Distribution of transmission classification of the cases depending on A) gender, B) the location of the cases.

b. Creation of the Outcome variable, merging Survival, ETU admission Status and Burial safety status

Three of the variables had interdependency: Survival, ETU admission status and Burial safety status: In the final dataset, every non-survivor admitted to an ETU was safely buried, and every survivor was admitted to an ETU. This could be due to a bias in detection for the people who stayed in the community and survived the outbreak. These individuals were less likely be reported to health authorities. In addition, no Survivor had a burial status (nor needs one). This imposes dependencies on the missingness in the data. Hence, we created Outcome, a compound variable with four levels, describing every combination observed in the dataset (Web Figure 4). Numbers observed in each class are given on the branches.

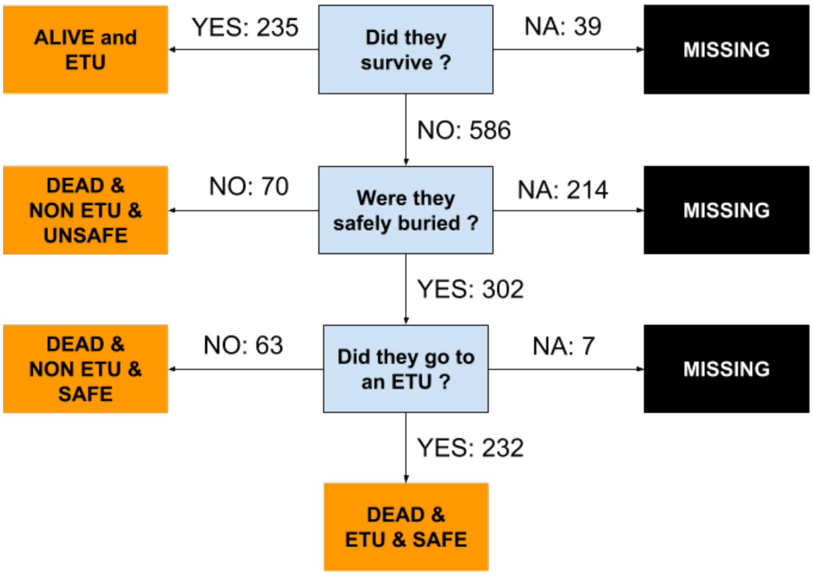

Web Figure 4: Creation of the compound variable Outcome, merging survival status, ETU admission status and burial safety status

c. Completeness of dataset

We observed an increase of the reporting of the burial safety status and ETU admission status through time (Web figures 5 and 6). From May 2015 onwards, almost all cases had their ETU admission status reported. We did not observe a clear change in the ETU admission rate through time, and almost the same proportion of cases stayed in the community in October 2014 and in May 2015. The fraction of cases admitted to an ETU during the last few months was much higher (after September 2015), but it involved only 2 cases. The fraction of safe burials was high throughout the outbreak (above 75%).

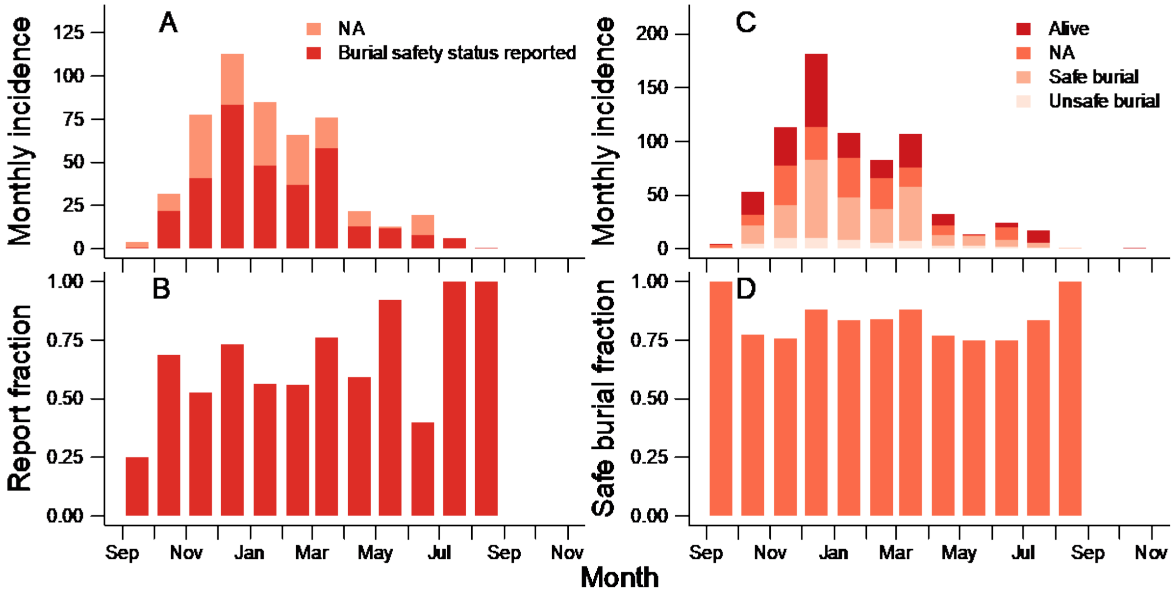

Web Figure 5: A) Monthly incidence of non-survivors, stratified by reporting of the burial safety status. B) Monthly report rate of burial safety status. C) Monthly incidence of the cases, stratified by burial safety status. D) Monthly rate of safe burial.

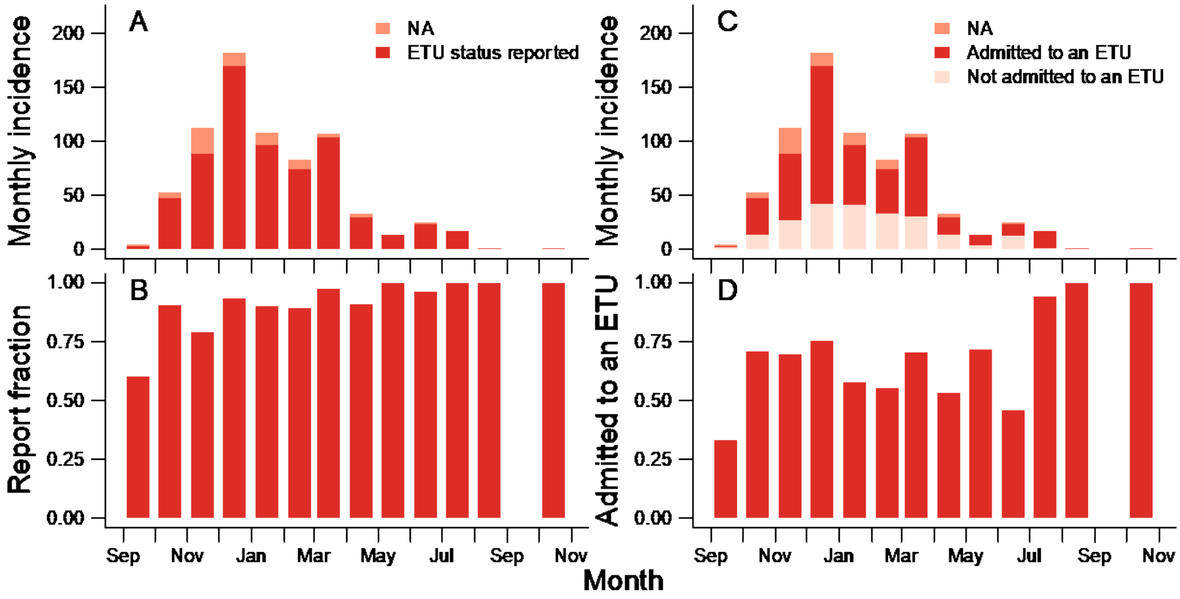

Web Figure 6: A) Monthly incidence of all cases, stratified by reporting of the ETU admission status. B) Monthly report rate of ETU admission status. C) Monthly incidence of all cases, stratified by ETU admission status. D) Monthly rate of ETU admission.

Web Appendix 4 - Multiple imputation of missing data

Four of the variables included in the negative binomial regression analysis were not fully reported: gender (1.7% missing), age (11.8% missing), survival status (4.5% missing), burial safety status (29.4% missing) and ETU admission status (13.6% missing). The latter three are included as the compound variable “Outcome” (section S3). 36.1% of the cases had at least 1 missing value. We used multiple imputation to generate plausible values and use the full dataset in the multivariate regression analysis (2,3). Using a rule of thumb to get a number of imputations superior to the percentage of missing cases, we created 40 imputed datasets(2). We performed the regression analysis on each imputed dataset and combined the estimates and standard errors using “Rubin’s rules”(2).

As explanatory variables, we included all other variables in the regression analysis (Number of secondary cases generated, Location, RVT status) and four fully reported auxiliary variables (Monthly incidence , number of community transmissions, funeral transmission and any nosocomial transmissions) (Web Table 1, Web Figure 7). The auxiliary variable “Monthly incidence” was computed using every indicator of timing (date of onset, date of ETU admission, date of death or discharge). We used the month of onset if the onset date was reported, otherwise we estimated the onset date from the other reported dates or those of the cases epidemiologically connected.

The multivariate imputation was performed with multiple imputation by chained equations (MICE package in R). We used different imputation method for each variable: Gender, survival status and ETU admission status all are two-level categorical variables and were imputed with a logistic regression model. We used conditional imputation on the burial safety status, as it had to be imputed only for dead individuals. As it had two possible imputed values (safe or unsafe), we used a logistic regression to impute the burial safety status (Web Table 1).

Imputed variables	Percentage missing (%)	Imputation method
Age	11.8	Predictive Mean Matching
Gender	1.7	Logistic
Survival status	4.5	Logistic
Burial safety status	29.4	Logistic
ETU admission status	13.6	Logistic
**Explanatory variables included in regression**	**Levels**	
Location	Urban / rural	
Secondary cases	Number of secondary cases generated per individual	
Generation number	Index of a chain / subsequent generations	
**Auxiliary variables**	**Description**	
Community transmission	Number of community transmissions caused per case	
Funeral transmission	Number of funeral transmissions caused per case	
Nosocomial transmission	Number of nosocomial transmissions caused per case	
Monthly incidence	Estimated month of onset	

Web Table 1: Description of variables included in the multiple imputation algorithm.

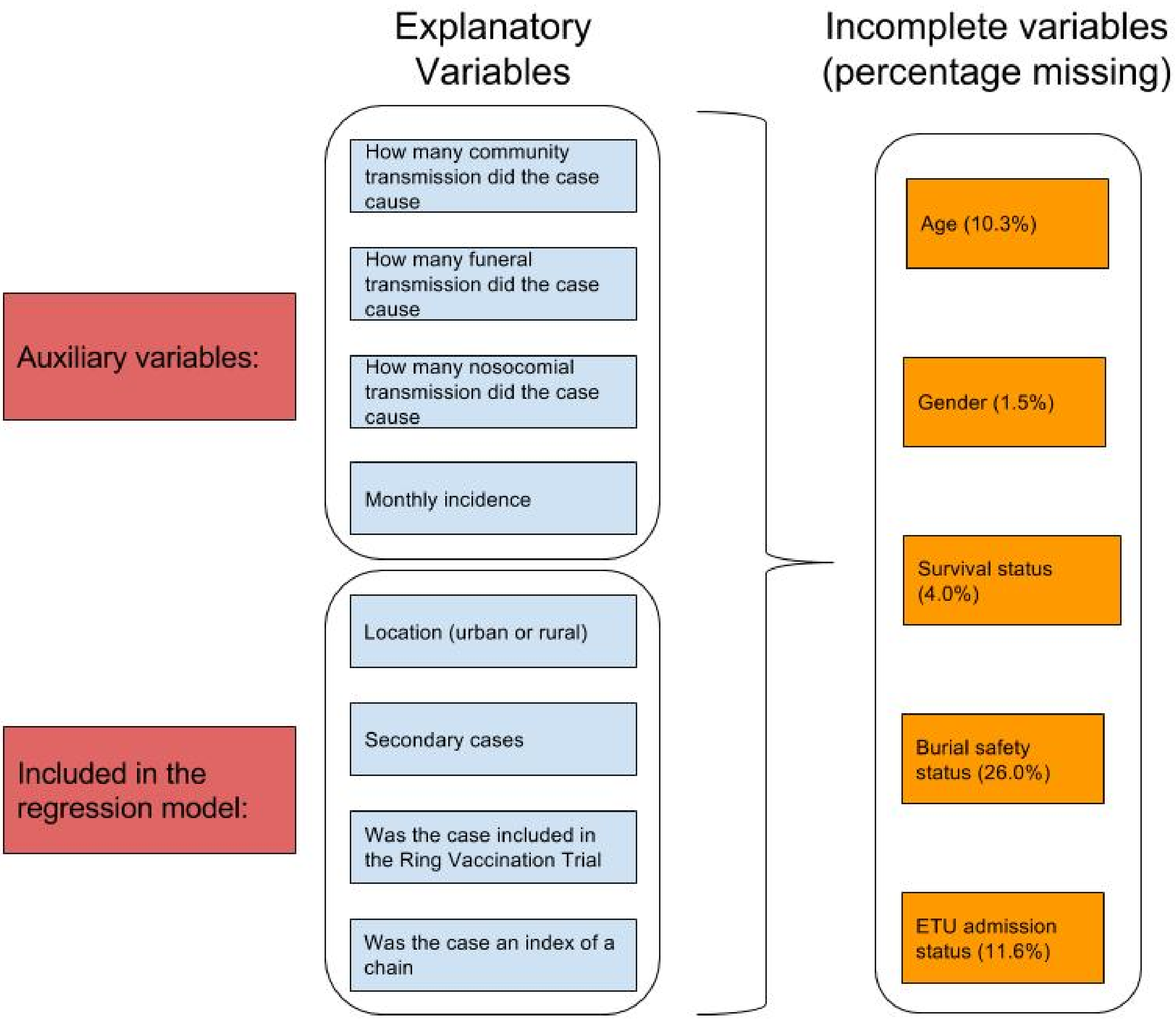

Web Figure 7: Description of the multiple imputation algorithm.

We considered the missing data were missing at random. The distribution of secondary cases for cases with missing values is not different than the one among complete cases (Web Figure 8). The cases with unreported survival status tend to be associated with lower number of secondary cases, it can also be due to the small sample size (39 cases with unreported survival status).

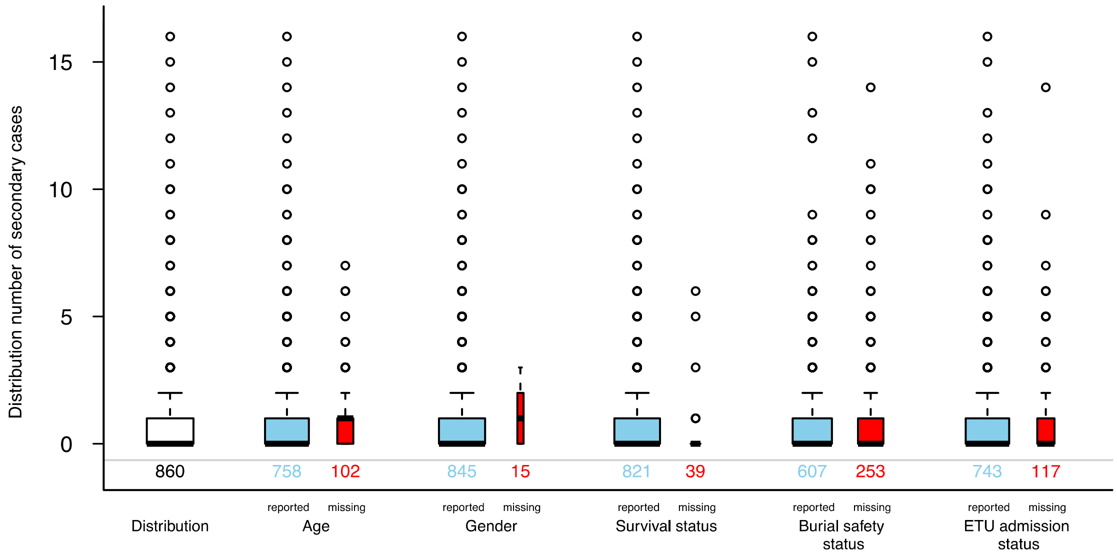

*Web Figure 8: Distribution of the number of secondary cases, depending on the reporting status of each variable. Numbers below each box show the number of cases in each category (i.e. age was reported for 758 cases). The outliers represent the values above 1.5*IQR.*

We observed different distributions of parameters for cases with unreported features, compared to the complete cases (Web Table 2). The cases with unreported features were almost exclusively non-survivors, which is due to the poor reporting of the burial safety status. Missing values tended to be observed among indexes of transmission chains. Comparison variable per variable did not show any difference in the distribution of secondary cases (Web Figure 9), but when the 311 cases with at least one missing entry are considered as a group, they are associated with a higher number of secondary cases, and were less likely to be admitted to an ETU. We performed every comparison using Kolmogorov-Smirnov test. This comparison shows that the data were not Missing Completely At Random, we considered they were Missing At Random (MAR) to perform the analysis.

Variables	Levels	Complete data (N=549) (%)	Incomplete cases (N=311) (%)	P value distribution
Age category	0-14	19.1	15.3	*P* = 0.33
	15-34	35.5	37.3	
	35-54	33.0	29.2	
	55-74	11.5	15.8	
	75-99	0.9	2.4	
Gender	Men	52.6	55.7	*P* = 0.99
	Women	47.4	44.3	
Survival status	Alive	41.3	3.0	$P < 10^{-3}$
	Dead	58.7	97.0	
Burial safety status	Survived	41.3	13.8	$P < 10^{-3}$
	Safe	9.3	32.8	
	Unsafe	49.4	53.4	
ETU admission status	No ETU admission	19.7	84.0	$P < 10^{-3}$
	Admitted to an ETU	80.3	16.0	
Location	Rural	45.9	66.2	$P < 10^{-3}$
	Urban	54.1	33.8	
Secondary cases	0	72.3	52.1	$P < 10^{-3}$
	1-3	22.2	37.6	
	4 and +	5.5	10.3	
Generation number	First generation	9.5	26.0	$P < 10^{-3}$
	Subsequent	90.5	74.0	
Community transmission	0	79.6	66.2	$P < 10^{-3}$
	1-3	16.3	29.0	
	4 and +	4.1	4.8	
Funeral transmission	0	97.6	92.6	*P* = 0.70
	1-3	2.0	7.0	
	4 and +	0.4	0.4	
Nosocomial transmission	0	97.4	94.9	*P* = 0.99
	1-3	2.2	5.1	
	4 and +	0.4	0	

Web Table 2: Epidemiological description of cases with complete reporting and cases with missing features

The imputed datasets have different distributions for some of the imputed variables (Web Figure 9). As most of the imputed cases did not survive nor went to an ETU, the imputed burial safety status was shifted towards “unsafe burial”. Age and gender distribution were not changed.

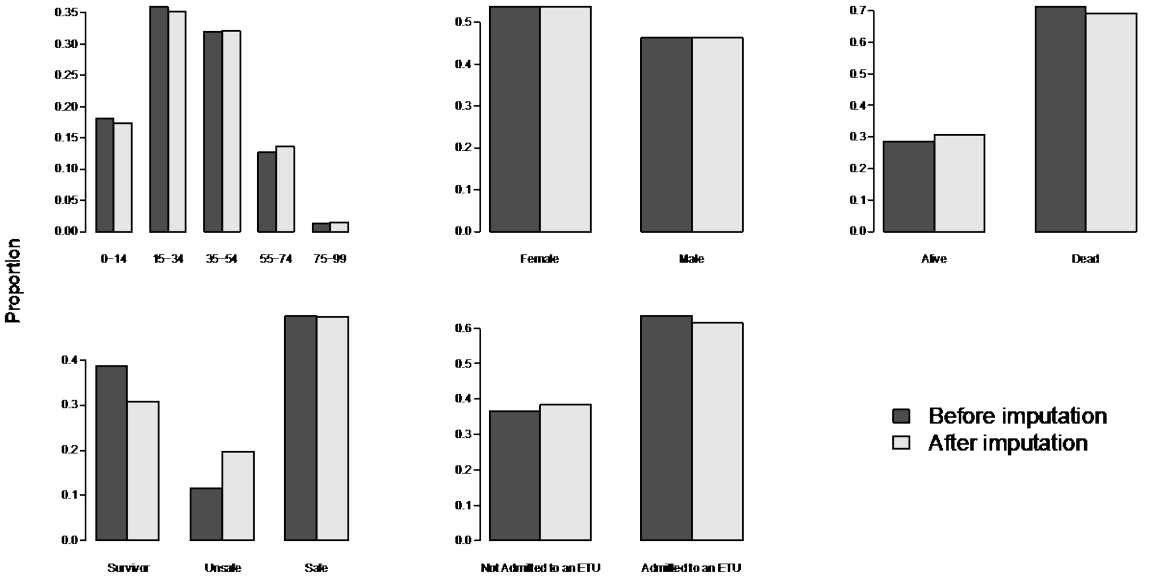

Web Figure 9: Distribution of variables with missing values, before and after imputation

For each imputed variable, the imputed values of the 40 datasets converged towards the same mean and standard deviation. As no dataset converge towards different value, we can perform the regression algorithm on all forty of them and pool the estimates.

Web Appendix 5 - Regression analysis and sensitivity

We used a negative binomial regression to analyse the imputed dataset. In our model, the estimated intercept accounts for the mean number of secondary cases for women, aged 35-54, who did not survive, did not go to an ETU and had a safe burial, in urban area and were not index of a chain. The full equation of the model is displayed in (1):

(1) $$\log(Sec) = Intercept + b1 * (Gender = \text{"}Male\text{"}) + b2 * (Outcome = \text{"}Alive, ETU+\text{"}) + b3 * (Outcome = \text{"}Dead, ETU+, safe\ burial\text{"}) + b4 * (Outcome = \text{"}Dead, ETU-, unsafe\ burial\text{"}) + b5 * (Location = \text{"}Rural\text{"}) + b6 * (Age = \text{"}0-14\text{"}) + b7 * (Age = \text{"}15-34\text{"}) + b8 * (Age = \text{"}55-74\text{"}) + b9 * (Age = \text{"}75-99\text{"}) + b10 * (Generation = \text{"}Index\text{"})$$

We modified the reference values in order to highlight the significance of the difference between survivors and non-survivors who attended an ETU. We defined the new intercept as the mean number of secondary cases for women, aged 35-54, who did not survive, went to an ETU and had a safe burial, in urban area and were not index of a chain. Survivors caused significantly fewer cases than non-survivors who got admitted to an ETU (Web Table 3).

		Secondary cases	IRR	95% CI	p-value
**Intercept**		0.43		0.25, 0.72	0.002
**Gender**	Female		*Ref*		
	Male		0.71	0.55, 0.93	0.012
**Outcome**	Alive, ETU+		0.51	0.31, 0.82	0.005
	Dead, ETU+, safe burial		*Ref*		
	Dead, ETU-, safe burial		1.63	1.01, 2.63	0.046
	Dead, ETU-, unsafe burial		2.97	1.97, 4.46	$P<10^{-3}$
**Urban/Rural**	Urban		*Ref*		
	Rural		1.18	0.90, 1.54	0.22
**Age**	0-14		0.35	0.21, 0.57	$P<10^{-3}$
	15-34		0.68	0.49, 0.93	0.015
	35-54		*Ref*		
	55-74		0.94	0.63, 1.40	0.76
	75-99		1.47	0.55, 3.91	0.44
**Generation number**	Subsequent generations		*Ref*		
	First generation		1.76	1.27, 2.44	0.001

Web Table 3: Results of regression analysis after we changed the reference levels. Here the intercept represents the mean number of secondary cases for women, who did not survive, went to an ETU and were safely buried, in urban area, aged 35-54 and from subsequent generation of a chain.

As 4.4% of the cases had several indexes, we drew among the possible infectors which one was considered in the analysis. We performed a sensitivity analysis to make sure this random allocation did not have any impact on the conclusion. We drew different infectors for these cases, and performed the multiple imputation algorithm on the new dataset. The results of the regression remained unchanged (Web Table 4).

		Secondary cases	IRR	95% CI	p-value
**Intercept**		0.43		0.25, 0.72	p<.05
**Gender**	Female		*Ref*		
	Male		0.74	0.57, 0.96	p<.05
**Outcome**	Alive, ETU+		0.52	0.33, 0.89	p<.05
	Dead, ETU+, safe burial		*Ref*		
	Dead, ETU-, safe burial		1.70	1.06, 2.73	p<.05
	Dead, ETU-, unsafe burial		2.89	1.93, 4.33	p<.001
**Urban/Rural**	Urban		*Ref*		
	Rural		1.10	0.84, 1.44	0.47
**Age**	0-14		0.36	0.22, 0.59	p<.001
	15-34		0.65	0.47, 0.91	p<.05
	35-54		*Ref*		
	55-74		0.93	0.63, 1.39	0.73
	75-99		1.33	0.52, 3.41	0.55
**Generation number**	Subsequent generations		*Ref*		
	Index of a chain		1.79	1.29, 2.49	p<.001

Web Table 4: Results of regression analysis after drawing an alternative set of infectors for cases with several potential infectors and performing the multiple imputation algorithm.

Web Appendix 6 – Additional characteristics of the dataset

We computed 308 serial intervals, describing the number of days between the date of onset of the index and the infectee. We did not observe any difference in duration depending on the age of the infected individual, nor on the route of transmission (Web Figure 10). We could not detect any impact of age of infectee or the route of transmission on the duration of the serial interval. The distribution of serial intervals of transmission events between the first and the second generation of the chains was similar to the distribution of subsequent transmissions, although the latter had a lower mean (11.8 days, and 14.5 days for the primary transmissions). Using the Kolmogorov Smirnov test to compare the distributions, we observed it was close to being significant (pvalue = 0.06).

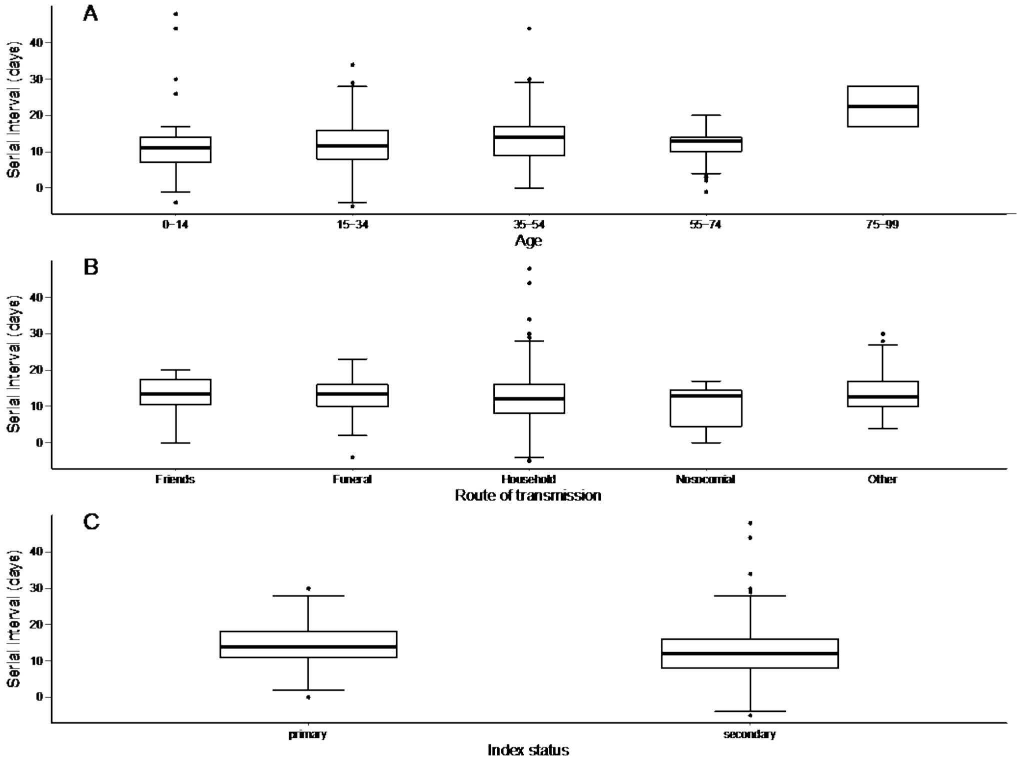

Web Figure 10: Comparison of the duration of serial interval, depending of A) Age of the infected individual, and B) Route of transmission, C) Primary or subsequent generations.

We fitted a negative binomial distribution to the number of secondary cases per infected individual in our dataset. We separated the indexes of the chains from the rest of the cases and observed the differences in distribution. The 133 indexes caused significantly more cases than the subsequent cases (Web Figure 11). The parameters of the fitted negative binomial distributions were significantly different (indexes: Mean =1.77, sd = 0.88 (95%CI: 0.53, 1.23), subsequent generation: Mean = 0.70, sd=0.25 (95%CI : 0.19, 0.30), Kolmogorov-Smirnov test $P<10^{-3}$).

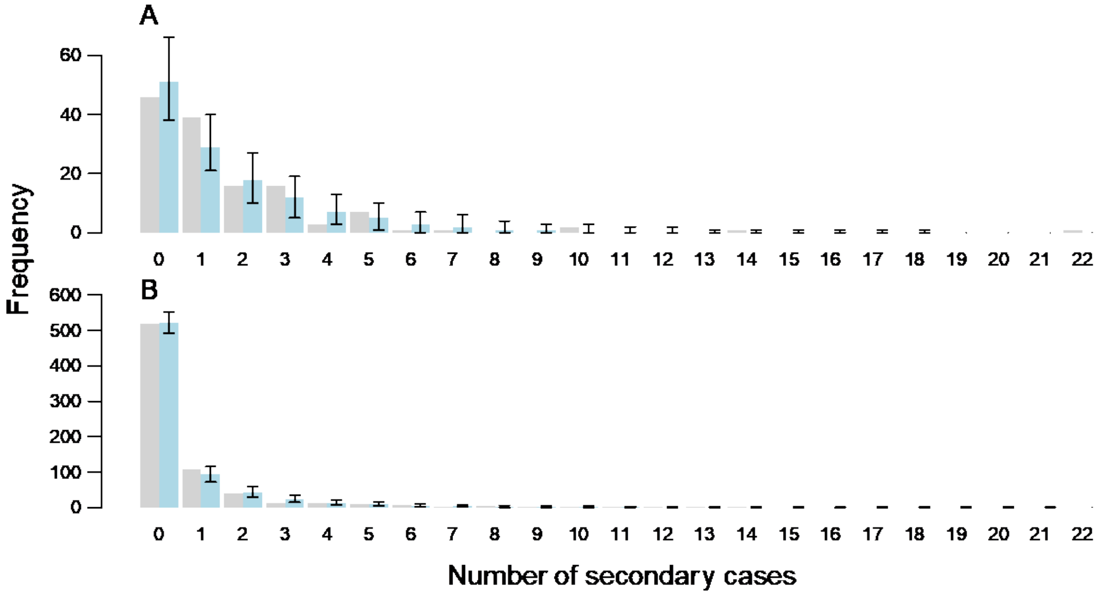

Web Figure 11: Distribution of the number of secondary cases generated per case, and fit to a negative binomial distribution (blue bars), displayed with 95% confidence intervals, for the A) Index of a chain (first generation) and B) Subsequent generations.

References

1. WHO. Ebola situation reports: Democratic Republic of the Congo [Internet]. 2018. Available from: https://www.who.int/ebola/situation-reports/drc-2018/en/

2. Donald B. Rubin. Multiple imputation for nonresponse in surveys. J.Wiley and Sons; 1987.

3. Sterne JAC, White IR, Carlin JB, Spratt M, Royston P, Kenward MG, et al. Multiple imputation for missing data in epidemiological and clinical research: potential and pitfalls. Bmj. 2009;
